# Genetic control of tolerance to drought stress in soybean

**DOI:** 10.1186/s12870-022-03996-w

**Published:** 2022-12-28

**Authors:** Aamir Saleem, Isabel Roldán-Ruiz, Jonas Aper, Hilde Muylle

**Affiliations:** 1 Flanders Research Institute for Agriculture, Fisheries and Food (ILVO), Plant Sciences Unit, Caritasstraat 39, 9090 Melle, Belgium; 2 Department of Plant Biotechnology and Bioinformatics, Ghent University, Technologiepark 927, 9052 Ghent, Belgium; 3 Protealis, Technologiepark-Zwijnaarde, Ghent, Belgium

**Keywords:** *Glycine max*, Genome-wide-association (GWAS), Quantitative trait locus (QTL), Single nucleotide polymorphism (SNP)

## Abstract

**Background:**

Drought stress limits the production of soybean [*Glycine max* (L.) Merr.], which is the most grown high-value legume crop worldwide. Breeding for drought tolerance is a difficult endeavor and understanding the genetic basis of drought tolerance in soybean is therefore crucial for harnessing the genomic regions involved in the tolerance mechanisms. A genome-wide association study (GWAS) analysis was applied in a soybean germplasm collection (the EUCLEG collection) of 359 accessions relevant for breeding in Europe, to identify genomic regions and candidate genes involved in the response to short duration and long duration drought stress (SDS and LDS respectively) in soybean.

**Results:**

The phenotypic response to drought was stronger in the long duration drought (LDS) than in the short duration drought (SDS) experiment. Over the four traits considered (canopy wilting, leaf senescence, maximum absolute growth rate and maximum plant height) the variation was in the range of 8.4−25.2% in the SDS, and 14.7−29.7% in the LDS experiments. The GWAS analysis identified a total of 17 and 22 significant marker-trait associations for four traits in the SDS and LDS experiments, respectively. In the genomic regions delimited by these markers we identified a total of 12 and 16 genes with putative functions that are of particular relevance for drought stress responses including stomatal movement, root formation, photosynthesis, ABA signaling, cellular protection and cellular repair mechanisms. Some of these genomic regions co-localized with previously known QTLs for drought tolerance traits including water use efficiency, chlorophyll content and photosynthesis.

**Conclusion:**

Our results indicate that the mechanism of slow wilting in the SDS might be associated with the characteristics of the root system, whereas in the LDS, slow wilting could be due to low stomatal conductance and transpiration rates enabling a high WUE. Drought-induced leaf senescence was found to be associated to ABA and ROS responses. The QTLs related to WUE contributed to growth rate and canopy height maintenance under drought stress. Co-localization of several previously known QTLs for multiple agronomic traits with the SNPs identified in this study, highlights the importance of the identified genomic regions for the improvement of agronomic performance in addition to drought tolerance in the EUCLEG collection.

**Supplementary Information:**

The online version contains supplementary material available at 10.1186/s12870-022-03996-w.

## Introduction

Human population growth and climate change are major challenges for the world’s future food security [1, 2]. Incidence of extreme weather events, such as erratic rainfall, raising temperatures and the consequent higher chance of drought spells cause significant reductions in crop production [3]. This, in combination with the need to feed a growing population without increasing the environmental burden on natural ecosystems, requires innovative solutions in agriculture, including the selection of crops with improved tolerance to water deficit through breeding [4]. Soybean [*Glycine max* (L.) Merr.] is the most grown-high value legume crop, and delivers a large part of the increasing demand for protein and oil in the world [5]. Drought stress has been identified as one of the challenges limiting soybean production in different parts of the world [6–9]. Therefore, breeding soybean for drought tolerance is imperative to avoid yield losses [10].

Drought stress causes several physiological, biochemical and molecular changes in plants such as reduced photosynthesis [11], oxidative stress caused by the accumulation of reactive oxygen species (ROS) [12], alterations in the metabolism of enzymes and other cellular compounds [13] and transcriptional modifications [14]. These changes are reflected in traits linked to crop performance including reduced stem growth and plant height, leaf wilting and senescence, altered root growth and reduced yield [15–17]. Our knowledge of drought tolerance in crops is fragmentary, but it is known that multiple plant characteristics and genomic loci influence the ability of plants to withstand drought stress [10, 18–20]. Furthermore, the timing, duration and intensity of the drought stress situation can strongly influence the plant responses [21–24]. Therefore, understanding the genetic basis of drought tolerance is crucial to get a better insight in tolerance mechanisms and for harnessing the genomic regions involved for crop improvement [25–27].

The genetic control of drought tolerance has been studied in major crops and, in some cases, this knowledge has been implemented in breeding programs. For example, in wheat, genes behind the synthesis and regulation of different types of enzymes and compounds such as Late Embryogenesis Abundant (LEA) responsive to abscisic acid (Rab), RuBisCO, helicases, proline, glutathione-S-transferase (GST) and carbohydrates are involved in drought tolerance [28]. QTLs have also been identified for water use efficiency (WUE) assessed through the carbon isotope ratio (δ^13^C), concentration of water-soluble carbohydrates (WSC), root system properties, and grain yield recorded under water stress [18]. Furthermore, [29] reported the introgression of multiple QTLs related to canopy temperature, chlorophyll content, stay green habit, Normalized Difference Vegetation Index (NDVI) values, days to anthesis and grain yield through marker assisted backcross breeding in wheat. Studies in maize have mainly focused on identifying the genetic basis of seedling survival rate, interval from anthesis to silking and yield-related traits [30]. Maize genotypes introgressed with QTLs for a short anthesis to silking interval outperformed for yield under drought but the yield advantage decreased from severe to mild stress. This illustrates that drought may activate different molecular mechanisms depending on the intensity of the stress situation [31]. Drought tolerance in rice has been assessed through screening for leaf rolling, spikelet fertility, rooting depth, leaf relative water content and osmotic adjustment [32], and a number of QTLs underlying some of these traits have been used for genomics-assisted breeding. For example, two drought tolerance QTLs (qDTY2.2 and qDTY4.1) have been introgressed into the popular, high-yielding rice variety IR64 [33], and three QTLs (qDTY1.1, qDTY2.2 and qDTY4.1) have been introgressed into the Indian elite variety Nareen [34]. These introgressions confer yield advantage under drought, and no penalty under non-stress conditions [33, 34].

The genetic control of drought tolerance has also been studied in soybean. Canopy witling is one of the most often investigated traits in soybean in the context of adaptation to drought. Correspondingly, several QTLs have been reported along with candidate genes putatively involved in the regulation of transpiration and water conservation [35–40]. Also QTLs for canopy temperature [41, 42], plant height in drought relative to control treatments [43, 44] and yield-related traits [45–47] have been reported. Overall, it has been shown that drought tolerance in soybean is conferred by many QTLs, and most of the QTLs explain only a little part of the phenotypic variation [20].

Previous studies of drought tolerance in soybean mainly focused on the analysis of late maturing soybean genotypes (MG0-IV or later). It is therefore unclear whether the same kind of adaptations and genomic regions are of relevance for exploitation in early maturing types (MG000-II). These early maturing types are however of particular importance for European agriculture [48], where there is a growing interest in the cultivation of soybean, as one of the approaches to increase protein self-sufficiency [49]. Considering that the local soybean cultivation accounts for only 34% of the total 34.4 million tons consumed in Europe [50, 51], increasing the European soybean acreage can significantly contribute to reduce protein imports. This requires breeding soybean varieties well-adapted to the European environmental and cultivation conditions. While North West Europe is often considered a humid region, simulations have demonstrated that water can be the main limiting factor in soybean production, because in general water availability is not well distributed along the crop cycle [52]. In addition, drought spells associated with low rainfall and high temperature are becoming more frequent in Europe during the summer [53–55], and forecasts on climatic change predict that the frequency of these events will increase in the future [56]. It is therefore important to consider adaptation to drought in European soybean breeding programs.

Drought spells occurring at the developmental stages following flowering are critical in soybean. At these stages soybean plants require sufficient water to achieve their yield potential [57], and investigations on drought tolerance in soybean focus on this [58–60]. In previous work [23] we investigated the response to drought at the reproductive stage of a collection of early maturing soybean accessions (MG000-II) of relevance for breeding in Europe (the EUCLEG collection). Responses and traits of relevance for adaptation to drought, and their interactions, were identified. Furthermore, we demonstrated a differential response of the accessions to short duration and long duration drought stress [23]. The EUCLEG collection displayed a wide range of phenotypic variation in drought response for maximum absolute growth rate (AGRmax), maximum canopy height (CH), leaf senescence (LSEN) and canopy wilting (CW). The long duration drought treatment (for 6–7 weeks) caused a much stronger response than the short duration drought (for 3–4 weeks). Main responses were an average reduction of 11–29% in CH, an average reduction of 22% in AGRmax and an acceleration of the rate of senescence by 26–110%. Drought stress accelerated the plant development. A better tolerance for seed yield was associated with an earlier cessation of flowering and pod formation under short duration drought stress, and with the maintenance of AGRmax and CH under long duration drought stress. Stronger signs of LSEN and CW helped also some accessions to protect their yield under long duration drought stress. This study provided a set of traits (AGRmax, CH, LSEN and CW) that might be used to improve drought tolerance of early maturing European soybean, what justifies a further exploration of the genetic control of these traits.

Genome-wide association (GWAS) is a widely used approach to study the genetic basis of phenotypic variation in crops [61], and several QTLs for important traits have been identified through GWAS in soybean [40–42, 62, 63]. A prerequisite for this is the availability of high-resolution genotypic data. As reported in [64], the EUCLEG collection has been genotyped using the NJAU 355K SoySNP array and a detailed genetic diversity analysis on the basis of 225K polymorphic SNPs (Single Nucleotide Polymorphisms) has been performed.

Here we build further on these two studies [23, 64] and report on a GWAS analysis on drought tolerance in the EUCLEG soybean collection. Our main goal is to understand the genetic control of drought tolerance in this collection of early maturing types. More specifically, we aim for the identification of (i) SNPs and QTLs related to drought response for canopy wilting, leaf senescence, maximum absolute growth rate and maximum plant height and (ii) the candidate genes underlying these QTLs and (iii) mechanisms of tolerance under short duration and long duration drought stress.

## Results

The results are based on separate analyses for the data collected in 2018 and 2019 because of the different weather conditions for these two years, causing a differential response of the accessions (for further details see [23]). For simplicity, here we refer to the experiments of 2018 and 2019 as the short-duration drought stress experiment (SDS) and the long-duration drought stress experiment (LDS) respectively.

### Variation in the drought response

**Fig 1.**
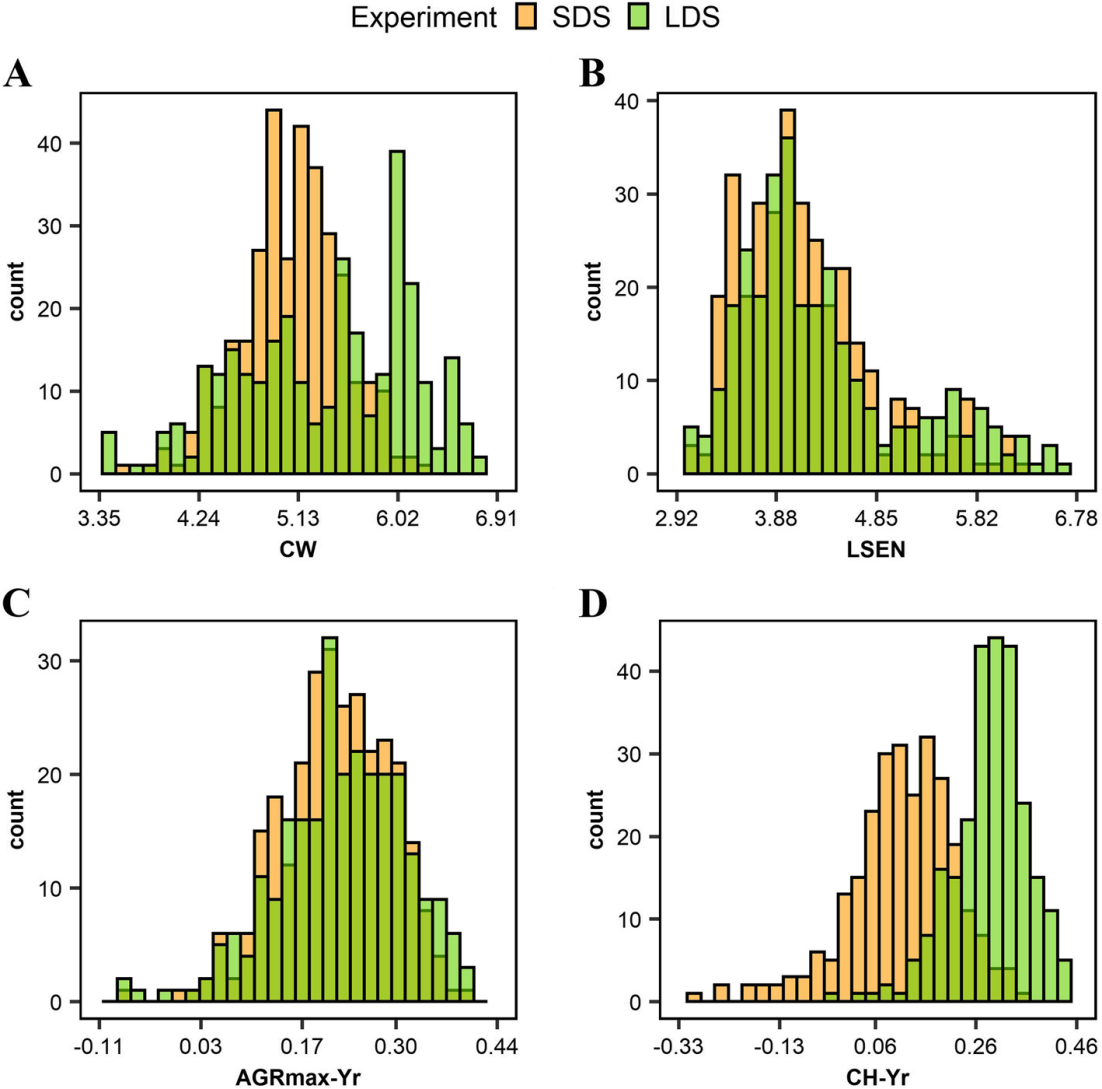
Distribution of the phenotype of Canopy wilting (**A**), Leaf senescence (**B**), Drought index for the maximum absolute growth rate (**C**) and Drought index for the maximum canopy height (**D**). Different colors indicate the short duration stress experiment (SDS) and the long duration stress experiment (LDS)

Basic statistics for the data of the four traits considered in this study (CW, LSEN, AGRmax-Yr and CH-Yr) are summarized in Table 1 and Figure 1. In general, the response to drought was stronger in the long-duration stress experiment (LDS), as indicated by higher average and maximum values recorded for the traits investigated, except for AGRmax-Yr (Table 1). Also the coefficient of variation was higher for all traits in the LDS experiment, except for CH-Yr (CV 24.30% in LDS and 25.20% in SDS). AGR-Yr and CH-Yr data displayed a normal distribution, while in the CW and LSEN data followed a slightly bi-modal distribution (Figure 1). This was the most pronounced in CW data recorded in the LDS experiment. These data indicate the presence of sufficient variation for the four traits investigated, and show that the data is suitable to use in GWAS analysis.

**Table 1:** Summary statistics of the traits considered in the GWAS analysis (adapted from [23])

Trait	Experiment*	Minimum	Maximum	Mean ± SD	CV%
Canopy wilting (CW)	SDS	3.58	6.20	5.08 ± 0.45	8.40
	LDS	3.35	6.91	5.38 ± 0.79	14.70
Leaf senescence (LSEN)	SDS	3.05	6.23	4.17 ± 0.64	12.90
	LDS	2.92	6.78	4.30 ± 0.81	16.10
Drought index for maximum absolute growth rate (AGRmax-Yr)	SDS	-0.08	0.39	0.22 ± 0.08	25.20
	LDS	-0.11	0.44	0.22 ± 0.09	29.70
Drought index for maximum canopy height (CH-Yr)	SDS	-0.3	0.35	0.11 ± 0.11	25.20
	LDS	-0.02	0.46	0.29 ± 0.08	24.30

^*^ *SDS* short-duration drought stress for a period of 3-4 weeks after initiation of flowering in 2018, *LDS* long-duration drought stress for a period of 6-7 weeks after initiation of flowering in 2019

### Genome wide association analysis

**Fig 2.**
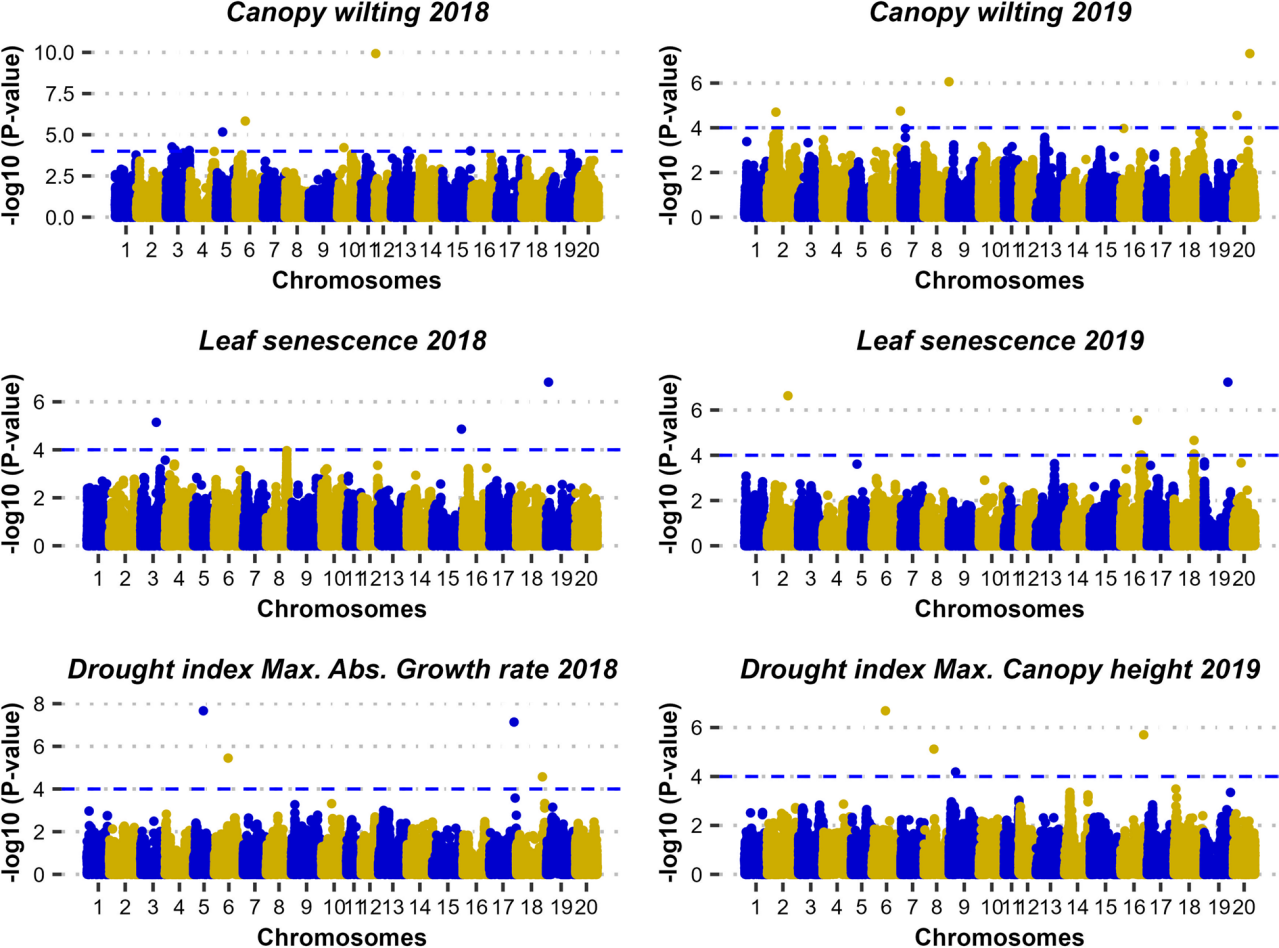
Manhattan plots of the GWAS analysis. The dashed horizontal line in blue indicates the threshold for -log_10_ *P* value ≥ 4. 2018 and 2019 in the plot’s titles represent ‘the short-duration stress experiment’ and ‘the long-duration stress experiment’ respectively

The results of the GWAS analysis are shown in Additional file 1, and the Q-Q plots are presented in Additional file 2. GWAS identified 17 and 22 significant marker-trait associations (-logP ≥ 4) in the SDS and LDS experiments respectively (Figure 2). No single SNP-trait association was consistent over the two experiments, indicating that different genomic regions might be relevant for stress tolerance under short-duration and long-duration drought stress.

A total of 471 and 508 genes of interest were identified in the candidate regions delineated around the significant SNPs in the SDS and LDS experiments respectively (Additional file 3). A set of 12 and 16 of these genes (in the SDS and LDS experiment respectively) carried annotations for their functions of particular relevance for drought (Table 2). Furthermore, several previously reported QTLs targeting important traits, including some related to drought tolerance, co-located with the candidate regions (Table 2 and Additional file 4).

**Table 2:** SNPs significantly associated with the four traits, genes of interest and previously reported QTLs in proximity of the significant SNPs. ‘Locus ID’ consists of the trait abbreviation followed by the name of experiment and an ordinal number per trait and experiment. ‘Candidate region’ consists of the chromosome ID followed by the start position and end position of the candidate region (defined by the LD decay distance around the significant SNP). ‘SNP ID’ consists of the ID of the significant SNP followed by the major allele and the minor allele of the significant SNP. ‘P’ is -log_10_ of the P-value of the significant SNP. ‘R^2^’ is Phenotypic variance explained by the significant SNP. ‘Allele effect’ is the difference between median trait value of genotypes carrying the major allele and minor allele. ‘Gene of interest’ refers to the gene of interest as in the reference genome Wm82.a2.v1. 'Arabidopsis' is the homolog gene ID and gene name (in parenthesis) from *Arabidopsis thaliana*. ‘Annotations’ is the gene transcript of the *Arabidopsis* homolog or its encoding protein retrieved from [65], and the biological function (in parenthesis). ‘Reference’ contains the literature references to the biological functions stated in 'Annotation'. ‘Known QTL’ contains QTL information retrieved from [66] [63] and [62]. The QTLs names are adapted. The original names of QTLs can be found in Additional file 4

Locus ID	Candidate region	SNP ID	P	R^2^	Allele effect	Gene of interest	Arabidopsis	Annotation	Reference	Known QTL
CW_SDS_1	Gm03_7027565_7317565	AX-93988673_G_A	4.3	0.05	-0.3	N/A	N/A	N/A	N/A	N/A
CW_SDS_2	Gm03_16874434_17164434	AX-93991327_G_C	4.1	0.02	0.23	N/A	N/A	N/A	N/A	N/A
CW_SDS_3	Gm03_45259915_45549915	AX-93918502_C_A	4.1	0.07	-0.4	Glyma.03G261500	AT2G46820 (*PSI-P*)	Photosystem I P subunit (photosynthesis)	[67, 68]	PubC 2, PubC 3
						Glyma.03G261400	AT4G01410 (N/A)	LEA hydroxyproline-rich glycoprotein (lateral root formation)	N/A	N/A
						Glyma.03G258300	AT3G61830 (ARF18)	Auxin response factor 18 (regulates cell growth and seed weight)	[69]	N/A
CW_SDS_4	Gm05_28547428_28867428	AX-93921335_C_T	5.2	0.06	0.27	N/A	N/A	N/A	N/A	SCN 1
CW_SDS_5	Gm06_14605039_14955039	AX-93923547_T_C	5.8	0.04	-0.18	N/A	N/A	N/A	N/A	PH 1, PH 2
CW_SDS_6	Gm10_7752764_8022764	AX-94072904_A_G	4.2	0.05	-0.3	N/A	N/A	N/A	N/A	PH 3, PH 4
CW_SDS_7	Gm12_268639_738639	AX-94092624_A_G	9.9	0.08	-0.29	Glyma.12G009100	AT5G09680 (RLF)	Reduced lateral root formation (lateral root formation)	[70]	
CW_SDS_8	Gm13_30347735_30657735	AX-93643614_G_A	4	0.04	0.29	N/A	N/A	N/A	N/A	WUE 1
CW_SDS_9a	Gm15_50341499_50871499	AX-94142375_T_C	4	0.06	-0.38	Glyma.15G269200	AT2G32840 (N/A)	Proline-rich family protein (cell wall integrity and root elongation under drought)	[71]	Cu 1, Cu 2
CW_SDS_9b	Gm15_50369101_50899101	AX-93945641_A_G	4	0.06	-0.38					
CW_LDS_1	Gm02_8486923_8816923	AX-93976757_G_A	4.7	0.09	0.62	Glyma.02G094700	AT4G01470 (TIP1-3)	Tonoplast intrinsic protein 1;3 (transmembrane channels for water and small uncharged solutes)	[72]	N/A
						Glyma.02G094900	AT3G55330 (PPL1)	PsbP-like protein 1 (reapir of PSII damage)	[73]	N/A
						Glyma.02G097700	AT1G25480 (N/A)	Aluminium activated malate transporter family protein (stomatal opening)	[74]	N/A
CW_LDS_2	Gm06_50717337_51067337	AX-93628808_T_C	4.7	0.14	0.73	Glyma.06G321900	AT1G55670 (PSAG)	Photosystem I subunit G (satbility of photosystem I complex)	[75, 76]	Seedset 1, Seeds
CW_LDS_3	Gm08_47511280_47831280	AX-93761082_G_T	6.1	0.14	1.04	Glyma.08G365700	AT1G67080 (ABA4)	Abscisic acid (aba)-deficient 4 (stress-induced ABA accumulation)	[77]	WUE 2, WUE 3
CW_LDS_4	Gm20_5309031_5709031	AX-93900908_A_C	4.6	0.14	0.76	Glyma.20G037100	AT5G12330 (LRP1)	Lateral root primordium (LRP) protein-related (root development)	[78]	N/A
CW_LDS_5	Gm20_43251405_43651405	AX-93910009_G_T	7.3	0.04	0.6	Glyma.20G197600	AT2G29460 (GSTU4)	Glutathione S-transferase tau 4 (oxidative stress response)	[79, 80]	N/A
LSEN_SDS_1	Gm03_35362990_35652990	AX-93917720_G_A	5.1	0.15	-0.53	Glyma.03G138000	AT5G19450 (CPK8)	Calcium-dependent protein kinase 19 (ABA-mediated stomatal closure and ROS reduction)	[81]	P, Mg, Zn
LSEN_SDS_2	Gm15_49950789_50480789	AX-94142205_C_T	4.9	0.14	-0.43	Glyma.15G266500	AT1G79580 (SMB)	NAC (No Apical Meristem) domain transcriptional regulator superfamily protein (root cap development)	[82, 83]	Cu 1, Cu 2
LSEN_SDS_3	Gm19_2913123_3603123	AX-93886487_C_T	6.8	<0.01	-0.01	Glyma.19G027700	AT5G39360 (EDL2)	EID1-like 2 (ABA signalling and stress respnse)	[84]	SIFC 1
LSEN_LDS_1	Gm02_36400063_36730063	AX-93981961_T_A	6.6	0.14	0.54	Glyma.02G192700	AT5G04870 (CPK1)	Calcium dependent protein kinase 1 (ROS reduction and proline accumulation)	[85]	N/A
						Glyma.02G193000	AT5G06410	DNAJ heat shock N-terminal domain-containing protein (maintenance of seed shape and size at high ABA; AtDjA3)	[86]	N/A
LSEN_LDS_2	Gm16_25479906_26001906	AX-93852357_C_A	5.6	0.11	-0.6	N/A	N/A	N/A	N/A	N/A
LSEN_LDS_3	Gm16_29164663_29686663	AX-93853705_G_C	4	<0.01	-0.02	N/A	N/A	N/A	N/A	PodShat 1, PodShat 2, qP16*
LSEN_LDS_4a	Gm16_30272259_30794259	AX-93854127_A_T	4	<0.01	-0.14	Glyma.16G145800	AT3G54890 (LHCA1)	Photosystem I light harvesting complex 1 chlorophyll a/b binding protein (electron transport in photosynthesis)	[87]	eChl_T**
LSEN_LDS_4b	Gm16_30395381_30917381	AX-93650691_A_C	4	0.01	0.08					
LSEN_LDS_5a	Gm18_48141658_48501658	AX-94178903_C_G	4	0.02	-0.24	Glyma.18G202900, Glyma.18G203500	AT1G09950 (RAS1), AT3G51810 (EM1)	RESPONSE TO ABA AND SALT 1 (Salt and ABA sensitivity), Em-like protein GEA1 (stress induced ABA response through ABI5, ROS reduction)	[88–90]	SCN 2, Oil
LSEN_LDS_5b	Gm18_48144745_48504745	AX-94178905_G_A	4	0.02	-0.24					
LSEN_LDS_5c	Gm18_48157192_48517192	AX-93655968_A_C	4	0.02	-0.24					
LSEN_LDS_5d	Gm18_48205607_48565607	AX-93655972_C_T	4.1	0.02	-0.21					
LSEN_LDS_5e	Gm18_48257716_48617716	AX-93881368_A_C	4.1	0.02	-0.21					
LSEN_LDS_5f	Gm18_48277688_48637688	AX-93952219_T_C	4.7	0.02	-0.24					
LSEN_LDS_5g	Gm18_48322720_48682720	AX-93881396_G_T	4.1	0.02	-0.21					
LSEN_LDS_6	Gm19_44805252_45495252	AX-94195039_A_G	7.2	0.12	-0.49	Glyma.19G191700	AT5G03720 (HSFA3)	Heat shock transcription factor A3 (thermotolerance and drought tolerance in combination with *DREB2A*)	[91, 92]	AMIN, Seedset 2, PH 5, Pods, LeafArea, LeafWidth, StemEnd, Internod, interbrach 1, DTM, DFTM, Lod, Nod, TotalSeed
AGRmax-Yr_SDS_1	Gm05_31841551_32161551	AX-93720346_T_C	7.7	0.1	0.06	Glyma.05G128000	AT1G29930 (LHCB1.3)	Light harvesting chlorophyll a/b binding protein 1 (stomatal movement, drought response)	[93]	N/A
AGRmax-Yr_SDS_2	Gm06_19076651_19426651	AX-93730252_G_T	5.4	0.09	0.05	Glyma.06G204100	AT3G29575 (AFP3)	ABI5 binding protein 3 (negative regulator of ABA signalling)	[94]	WUE 4, WUE 5, interbrach 2, DTF
						Glyma.06G204400	AT1G74160	LONGIFOLIA protein (cellulose deposition)	[95, 96]	N/A
AGRmax-Yr_SDS_3	Gm17_39250897_39530897	AX-93866910_A_C	7.1	<0.01	0.01					
AGRmax-Yr_SDS_4	Gm18_54299774_54659774	AX-93883955_T_A	4.6	0.09	-0.04	Glyma.18G259700	AT5G15250 (FTSH6)	FtsH protease 6 (thylakoid membrane biogenesis, PSII repair mechanism)	[97]	SCN 3, Fe
CH-Yr_LDS_1	Gm06_18615861_18965861	AX-93730063_A_G	6.7	0.06	-0.03	Glyma.06G202200	AT1G74310 (HSP101)	Heat shock protein 101 (tolerance to heat sterss)	[98]	PubC 1, PubC 4
CH-Yr_LDS_2	Gm08_15065114_15385114	AX-93754190_C_T	5.1	0.16	0.06	Glyma.08G191300	AT4G33050 (EDA39)	Calmodulin-binding family protein (stomatal opening)	[99]	PUE, SIFC 2
CH-Yr_LDS_3	Gm09_7236144_7576144	AX-94060112_T_C	4.2	0.16	0.05	Glyma.09G071400	AT1G19150 (LHCA6)	Photosystem I light harvesting chlorophyll a/b binding protein 6 (photosynthesis)	[100]	WUE 6
CH-Yr_LDS_4	Gm16_32550790_33072790	AX-93855040_G_A	5.7	0.16	0.06	N/A	N/A	N/A	N/A	BSR

^*^ QTL information from [63]

^**^ QTL information from [62]

### Canopy wilting (CW)

Ten SNPs displayed significant associations with CW in the SDS experiment (Table 2). These SNPs were distributed over seven chromosomes, and explained individually 2−8% of the phenotypic variation (R^2^). The candidate genes present in the candidate genomic regions surrounding the significantly associated SNPs are putatively involved in lateral root formation, growth, photosynthesis and cell-wall integrity in *Arabidopsis* plants (Table 2). For example, the candidate region defined by SNP AX-93918502 contains Glyma.03G261500, annotated as a P subunit of Photosystem-I protein encoding gene (*PSI-P)* and Glyma.03G258300, annotated as *‘Auxin Response Factor 18 (ARF18)’* which is known to regulate cell growth and seed weight in *Arabidopsis*. Glyma.03G261400, also present in the candidate region surrounding SNP AX-93918502, is annotated as ‘LEA hydroxyproline-rich family protein gene’, which plays a role in lateral root formation in *Arabidopsis*. Glyma.12G009100, in the candidate region defined by SNP AX-94092624 is an *Arabidopsis* homolog of *‘Reduced Lateral root Formation (RLF)’*. The candidate region defined by SNP AX-93945641 contains Glyma.15G269200, an *Arabidopsis* homolog encoding a proline rich protein that has been shown to play a role in cell wall integrity and root elongation during drought stress in *Arabidopsis*. As shown in Table 2, SNP AX-93643614 collocates with an interesting QTL for water use efficiency (WUE).

For CW data recorded in the LDS experiment, five SNPs displayed significant associations. These SNPs were distributed over four chromosomes and explained individually 4-14% of the phenotypic variation (R^2^) for CW (Table 2). Among the genes identified in the candidate regions surrounding these SNPs, several are related to stress response, root formation, trans-membrane transport of water and solutes, stomatal movement, repair and maintenance of the photosynthetic apparatus and cellular protection through ROS scavenging. For example, the candidate region surrounding SNP AX-93976757 comprises Glyma.02G094700, annotated as ‘*TIP1-3’* that putatively encodes a member of tonoplast intrinsic aquaporins (transmembrane channels for water and small uncharged solutes), Glyma.02G094900, annotated as *‘PsbP-like protein 1 (PLP1)’* which functions to repair damage in Photosystem II in *Arabidopsis*, and Glyma.02G097700, annotated as an ‘*Aluminium activated malate transporter’*, having a putative role in stomatal opening. The candidate region defined by SNP AX-93761082 contains Glyma.08G365700, an *Arabidopsis* orthologue of *Abscisic acid-deficient 4 (ABA4)* which is involved in stress-induced ABA accumulation. Interestingly, two QTLs for WUE have been also reported in this region (Table 2). Glyma.20G037100 (on Gm20) is an *Arabidopsis* orthologue of *Lateral Root Primordium Protein-related* (*LRP1*) and Glyma.20G197600 (on Gm20) is an *Arabidopsis* orthologue of an antioxidant protein gene *‘Glutathione S-transferase tau 4 (GSTU4)’*.

### Leaf senescence (LSEN)

Three SNPs locating on three different chromosomes displayed significant associations with LSEN in the SDS experiment, and explained up to 15% of the phenotypic variation. The candidate regions defined by these SNPs contain genes putatively involved in stress response, response to ABA and root cap development. The most strong effect was detected for a candidate region surrounding SNP AX-93917720, that contains Glyma.03G138000, which is annotated as a *‘Calcium-dependent Protein Kinase 19 (CPK8)’* known to be involved in ABA-mediated stomatal closure and ROS reduction in *Arabidopsis* plants. In the candidate region surrounding SNP AX-94142205, Glyma.15G266500 is annotated as ‘*NAC* (*No Apical Meristem) domain transcriptional regulator*’ (*SMB*), whose *Arabidopsis* orthologue controls root cap development. The region surrounding SNP AX-93886487 contains Glyma.19G027700, which is related to *EID1-like 2*, a member of F-box proteins and a positive regulator of ABA signaling to produce responses such as control of germination and root growth in *Arabidopsis* plants under drought stress conditions.

The 13 SNPs that displayed significant associations with LSEN in the LDS experiment are distributed over six chromosomes and explain 1-14% of the phenotypic variation. The candidate regions surrounding these SNPs contain genes involved in photosynthesis, ABA signaling, cellular repair and ROS scavenging. For example, the candidate region defined by SNP AX-93981961 contains Glyma.02G192700, annotated as *‘Calcium-dependent Protein Kinase 1 (CPK1)’*, and Glyma.02G193000, related to ‘*AtDjA3, a* ‘DNAJ heat shock N-terminal domain-containing protein encoding gene’ in *Arabidopsis*. *CPK1* functions in proline accumulation and ROS reduction, while *AtDjA3* is known to maintain seed shape, seed size and seedling survival at high levels of ABA. The region between 25.5 Mb and 30 Mb on Gm16 contains four significant SNPs. A QTL (eChl_T) for total chlorophyll content has been reported for this region, and Glyma.16G145800, also present in this region is annotated as *‘LHCA1’* in *Arabidopsis* which encodes a photosystem I light harvesting complex chlorophyll a/b binding protein. The region between 48.1 Mb and 48.6 Mb on chromosome Gm18 contains seven significant SNPs, and contains two genes (Glyma.18G202900 and Glyma.18G203500) involved in salt and ABA responses and in ROS reduction*.* The candidate region defined by SNP AX-94195039 contains Glyma.19G191700 or *‘HSFA3’.* In *Arabidopsis,* this gene encodes a heat shock protein that plays a role in thermotolerance and drought tolerance in combination with *DREB2A*.

### Drought response for maximum absolute growth rate (AGRmax-Yr)

Only in the SDS experiment significant SNP-trait associations were found for AGRmax-Yr. These four SNPs were distributed over four chromosomes and explained up to 9% of the phenotypic variation. Genes putatively involved in stomatal movement, cell division, photosynthesis and response to ABA and stress response were contained in the delineated candidate regions. Glyma.05G128000 (orthologue of *LHCB1.3* in *Arabidopsis*) putatively encodes a ‘light harvesting Photosystem II chlorophyll a/b binding protein’ that regulates stomatal movement and plant responses to drought, while Glyma.18G259700 is an ortholgoue of *FtsH protease* that functions in thylakoid membrane biogenesis and Photosystem II repair in *Arabidopsis*. On Gm06, Glyma.06G204100 is annotated as ‘*ABI5 binding protein 3 (AFP3)’* which negatively regulates ABA response in *Arabidopsis*. Two previously reported QTLs for WUE are also present in the same region on Gm06. In addition, the same region is located in the neighborhood (at 4.1 Mb) of one candidate region for CW (CW_SDS_5) (Table 2).

### Drought response for maximum canopy height (CH-Yr)

For CH-Yr, significant SNP-trait associations were only detected in the LDS experiment. In this case four SNPs distributed over four chromosomes and explaining 6-16% of the variation displayed significant associations. The candidate genes identified in the neighborhood of these significant SNPs are putatively involved in stress response, stomatal movement and photosynthesis. Glyma.06G202200 is orthologue to ‘*HSP101’* which encodes a heat shock protein in *Arabidopsis*. On Gm08, Glyma.08G191300 is annotated as ‘calmodulin-binding family protein gene’ which is known to be involved in stomatal opening in *Arabidopsis*. On Gm09, Glyma.09G071400 (or ‘*LHCA6’* in *Arabidopsis*) encodes a ‘light harvesting Photosystem I chlorophyll a/b binding protein 6’. A previously reported QTL for WUE is also present in the same region on Gm09.

Some candidate regions for CH-Yr are located close to candidate regions for CW and LSEN. i.e. on Gm16 CH-Yr_LDS_4 is located next to LSEN_LDS_3, LSEN_LDS_4a and LSEN_LDS_ 4b with a maximum distance of 2.6 Mbp between them, and on Gm16, CH-Yr_LDS_1 is located next to CW_SDS_5 with 3.6 Mbp between them (Table 2).

## Discussion

### GWAS revealed novel loci associated with tolerance to a short duration and a long duration drought stress

The GWAS analysis identified a total of 17 and 22 significant marker-trait associations for four traits (CW, LSEN, AGRmax-Yr, CH-Yr) in the short-duration and long-duration drought experiments respectively. None of these SNPs was common to the two experiments. This was not completely unexpected because the overall phenotypic response of the EUCLEG soybean collection in two experiments was different [23]. A total of 12 and 16 of the genes that are in LD with these SNPs (for SDS and LDS experiments respectively) are of particular relevance for drought stress responses including stomatal movement, root formation, photosynthesis, ABA signaling, cellular protection and cellular repair mechanisms. Furthermore multiple previously reported QTLs related to drought tolerance traits such as WUE, chlorophyll content and photosynthesis, and QTLs for traits including plant height and other yield-related traits, disease tolerance, nutrient content, seed composition also co-localize with the significantly associated SNPs.

### Short duration and long duration drought treatment induce differential mechanisms of canopy wilting in soybean

Slow or delayed wilting is known to be a complex trait, that can be the result of several underlying mechanisms in soybean: (i) good water resource exploration by a large root system [101]; (ii) lower stomatal conductance, reduced transpiration rate and high water use efficiency [102]; (iii) maintenance of constant transpiration rate under vapor pressure deficit conditions above 2.0 kPa [103]; and (iv) lower radiation use efficiency [104]. Several of the candidate genes identified in the SDS experiment are putatively involved in lateral root development, and some are related to photosynthesis. Root system modification is an important response that shows a large phenotypic plasticity in soybean under drought stress [15]. This response is a drought avoidance mechanism enabling plants to sustain high plant water status or cellular hydration under drought [105]. Drought stress inhibits root growth in general but susceptible genotypes show more prominent effects [106, 107] due to an overall decrease in newly synthesized cell wall polysaccharides such as pectin, hemicellulose, and cellulose [108]. [107] discuss how the taproot length and tertiary root length influence the root surface area, what in turn influences the plant nutrient and water absorption capacity. Similarly, soybean genotypes with high root length, surface area, diameter and volume achieved high net photosynthesis, attained higher plant height and biomass, and tended to perform better under water deficit conditions [106]. The well-described slow wilting soybean accession PI 796397 has a dense root system with a high number of root tips [109]. We have shown a large level of variation for drought index for canopy height (CH-Yr) and number of pods per main stem (PPS-Yr) in the EUCLEG soybean collection [23]. Some accessions were able to maintain high CH and displayed no reduction in the number of pods per plant (NPP), but the relationship between these traits and CW was not clear. This might be due to compensation during the period of stress recovery as discussed in [23]. Anyhow, the results obtained in this work regarding marker-trait associations support the hypothesis that the mechanism of slow wilting in the SDS experiment might be associated with the characteristics of the root system.

In the LDS experiment, the associated SNPs were in LD with genes related to stress-induced abscisic acid (ABA) accumulation, stability of the photosynthetic apparatus, oxidative stress response and ROS scavenging, and trans-membrane water and solute transport, along with one gene related to root development. Some candidate regions in LDS also overlapped with previously reported QTLs for WUE [110]. In [23] we have reported a drastic reduction of the growth rate, plant height and yield in the LDS treatment. These plant responses match with the dehydration tolerance mechanism that is activated under extreme drought stress conditions [111]. Dehydration tolerant plants tend to maintain metabolic activities at low tissue water potential through osmotic adjustment, antioxidant activities, and altered growth regulators [112]. Soybean plants subjected to drought for 20 days at V5 stage in [113] showed a higher accumulation of ABA, lower stomatal conductance and decreased rate of net photosynthesis, but resistant genotypes performed relatively better than the susceptible ones. In [114], drought stress disturbed the balance between ROS and antioxidant enzymes, and a severe stress led to overproduction of ROS causing cellular damage, low stomatal conductance and a decrease in photosynthesis parameters in soybean. Drought tolerant soybean genotypes in [22] developed an anti-oxidative defense mechanism for ROS scavenging by increasing the activity of antioxidant enzymes including the peroxidase superoxide dismutase, ascorbate peroxidase, glutathione reductase and catalase, which helped them to maintain a high photosynthetic efficiency and a high RuBisCo activity under heavy stress conditions. Our results suggest that under severe stress, canopy wilting response in soybean is regulated by a different mechanism than under short duration stress. Based on the marker-traits associations identified in the LDS experiment, slow wilting genotypes can achieve a high WUE and thus a high level of tolerance to long-term drought stress through a reduction of stomatal conductance and transpiration rates.

### Drought-induced leaf senescence is related to ABA and ROS responses in soybean

GWAS identified 3 and 13 significant SNPs for LSEN in the SDS and LDS experiments respectively. Premature leaf senesce is thought to be an indicator of stress vulnerability in plants [115]. [116] described a negative correlation between premature leaf senescence and plant survival in a perennial temperate grass. According to the mechanism of premature leaf senescence, accumulation of ABA under stress conditions promotes overproduction of ROS which may react with proteins, lipids and deoxyribonucleic acid, leading to oxidative damage and premature leaf senescence [84, 85]. Several transcription factors that play a role in the control of age-induced leaf senescence, play also a role in plant stress tolerance, and chloroplast breakdown is a common feature in both cases [115]. Here we also identified multiple candidate genes for LSEN. Three candidate genes known to be involved in ABA-mediated stomatal closure, root cap development and ROS scavenging were identified in the SDS experiment. In the LDS experiment, six candidate genes had stress related functions such as ABA-mediated seedling survival, maintenance of seed shape and seed size at high ABA levels, drought and salt tolerance through proline accumulation and ROS scavenging, and stability of Photosystem I. In the work of [117] a higher WUE and an increased tolerance obtained through the activation of a cell wall invertase in tomato was associated with a low stomatal conductance, delayed senescence, increased source activity and a better control of ROS production under drought stress. In [118] minimizing the stress-mediated senesce by the over expression of a senescence associated gene enabled the plants to attain higher shoot and root biomass and to recover better after a period of drought stress. Our results also reveal that drought-induced premature senescence in soybean can be avoided by targeting the genes involved in ROS scavenging and stomatal conductance.

Furthermore, our results reveal that two candidate regions (LSEN_LDS_3 and LSEN_LDS_4a on Gm16) can be of particular significance as they co-localize with QTLs for net photosynthesis rate (qP16; [63]) and for total chlorophyll content (eChl-T; [62]). Moreover, the candidate gene Glyma.16G145800, having a putative role in chlorophyll synthesis [87], is also present in the candidate region LSEN_LDS_4a. It has been discussed that the drought-induced accelerated senescence affects source-sink relationship in wheat [119] and causes a significant yield reduction in soybean [120]. The candidate region LSEN_LDS_6 on Gm19 contains a gene encoding a heat shock protein which confers stress tolerance [91, 92]. The same region also contains many previously reported QTLs for important agronomic traits including leaf growth, plan height, internode length, number of pods and seed amino acid content, indicating the relevance of this candidate region to improve drought tolerance and agronomic performance in soybean.

### QTLs related to WUE play a role in the maintenance of growth rate and canopy height under drought stress

Our GWAS analysis identified also candidate genes for drought response of maximum absolute growth rate (AGRmax-Yr) and drought response of maximum canopy height (CH-Yr). These traits were determined using UAV-based methods [23], and quantify the growth of the soybean accessions in the drought treatment relative to the control treatment. AGRmax-Yr and CH-Yr are therefore comprehensive indicators of drought tolerance, and are derived by the actions of multiple physiological processes, including the rate of carbon assimilation, photosynthesis, respiration, source and sink relations, nutrient balances, cell differentiation and elongation, as well as belowground processes [43, 121, 122]. A total of four significant associations were identified by GWAS for AGRmax-Yr in the SDS experiment, and another four significant associations for CH-Yr in the LDS experiment. Allelic differences of the significant SNPs explained a low to high drought-induced reduction for maximum absolute growth rate and for maximum canopy height in the EUCLEG accessions. The comprehensive nature of these growth related traits was reflected in the fact that some of the candidate regions for AGRmax-Yr and CH-Yr are located closely to the other candidate regions for CW and LSEN (on Gm05, Gm06 and Gm16; Table 2).

Furthermore, two previously reported QTLs for WUE [110] coincided with the candidate region AGRmax-Yr_SDS_2, on Gm06. This region contains Glyma.06G204400, whose *Arabidopsis* orthologue *TRM4 or* LONGIFOLIA is considered essential for cellulose deposition [95], whereas cellulose is essential for stem growth [96]. The same region contains Glyma.06G204100, annotated as *Arabidopsis AFP3,* which is a negative regulator of ABA response as it causes proteomic degradation of ABI5 [94], a core element in ABA response [123]. Furthermore, another candidate region CH-Yr_LDS_1, on Gm06 contains Glyma.06G202200, annotated as *Heat-shock Protein 101* in *Arabidopsis* (*HSP101*). Constitutive expression of *HSP101* provided heat tolerance and a high survival rate without any detrimental effect on growth in *Arabidopsis* plants [98]. These two regions on Gm06 can be of particular interest for further research and to modulate drought tolerance in soybean.

SNPs in candidate regions CH-Yr-LDS_2, _3 and _4 explained a relatively large proportion of the phenotypic variation for CH-Yr (R^2^ = 0.16; Table 2). The candidate gene Glyma.08G191300 (*Arabidopsis EDA39*) on Gm08 belongs to the calmodulin-binding family protein. *EDA39,* in coordination with *AtWRKY21,* promotes stomatal opening by down-regulating the ABA response [124]. Another interesting candidate gene is Glyma.09G071400 (*Arabidopsis LHCA6*), present on Gm09. *LHCA6* was shown to play a role in the normal functioning of the chloroplast [125], and its reduced expression caused a lower photosynthesis in *Arabidopsis* plants [100].

Considering the high level of stress imposed in the LDS treatment, and the results presented above for canopy wilting in the LDS experiment, it is obvious that within the EUCLEG collection some accessions have mechanisms to minimize transpiration through stomatal regulation, ensuring survival under long-term stress treatment. Therefore, our results suggest that a high water use efficiency could be an effective means to improve drought tolerance of this European soybean collection, in particular for environments in which the chance of long periods of drought is high. Further, our results indicate that for an effective drought tolerance under long-term drought stress, modulating the genes related to photosynthesis could be promising in soybean.

## Conclusion

The present study applied a genome-wide association study (GWAS) analysis and investigated the genetic control of drought tolerance in the EUCLEG soybean collection relevant for breeding in Europe. The GWAS analysis identified a total of 17 and 22 significant marker-trait associations for four traits, in the short duration drought (SDS) and long duration drought (LDS) experiments, respectively. Based on these marker-trait associations a total of 12 and 16 genes of particular relevance for drought stress responses including the stomatal movement, root formation, photosynthesis, ABA signaling, cellular protection and cellular repair mechanisms, were identified. Several previously known QTLs for drought tolerance traits such as WUE, chlorophyll content and photosynthesis, co-localized with the significantly associated SNPs, suggesting their role in drought tolerance mechanisms in soybean. Our results revealed a differential mechanism of canopy wilting in the EUCLEG collection under SDS and LDS. The marker-trait associations in the SDS targeted multiple genes involved in root elongation, root cap development, lateral root formation etc., suggesting that slow canopy wilting in the SDS might be associated with the characteristics of root system. In the LDS, several of identified genes and previously known QTLs carried annotations related to water transport, maintenance of photosynthetic apparatus and cellular protection and WUE, suggesting that slow wilting response under the LDS might be associated with a high WUE through a reduction of stomatal conductance and transpiration rates. Our results also showed that the drought-induced leaf senescence could be related to ABA and ROS responses in soybean, and QTLs related to WUE might have played a role in the determination of growth rate and canopy height under drought stress. Many previously reported QTLs for multiple agronomic traits also co-localized with the significantly associated SNPs, suggesting that the identified genomic regions might have the potential to improve the agronomic performance together with the drought tolerance in the EUCLEG collection. These results provide an important basis to improve drought tolerance in soybean in Europe. The genomic regions identified in this study can be further explored to ensure their added value in the genomic assisted breeding i.e. genomic selection.

## Materials and methods

### Field trials and phenotypic evaluation

The set of 359 soybean accessions originating from 25 countries in Europe, China and the USA of [23] was used. This set is a part of the EUCLEG collection assembled in context of the EUCLEG project (www.eucleg.eu). It represents widely the germplasm available for breeding of soybean in Europe. They are the gene bank accessions from the ARS-GRIN database and the IPK database, and the accessions from the collaborators of the EUCLEG project. This set has been described in [23] and [64]. The accessions were divided into four groups (GP), based on maturity information [23]. Accessions in GP1 (*n*=90), GP2 (*n*=91), GP3 (*n*=88), and GP4 (*n*=90) were expected to belong to maturity groups MGI/II, MG0, MG00, and MG000, respectively [23]. This wide representation of genetic diversity of the EUCLEG collection makes it an ideal collection for evaluating its response to drought stress and subsequently for the GWAS analysis to identify the genomic loci affecting the drought tolerance of soybean.

These accessions were sown in 2018 and 2019 in Melle, Belgium (51.00° N, 3.80° E) on sandy loam soil. The experimental set-up has already been described in [23]. In short, each year (2018 and 2019) two adjacent fields sown. One of these fields was used as ‘control’, while the other was subjected to a ‘drought’ treatment. The four GPs were sown on four different dates (between 20 April and 11 May in 2018 and between 19 April and 15 May in 2019) to synchronize the developmental stage at which drought was imposed (i.e. when 50% of the plots had initiated flowering). The plot dimensions were slightly adjusted for four different GPs in order to implement their recommended row-to-row spacing and sowing densities (45, 55, 65, and 75 seeds m^-2^ for GP1, GP2, GP3, and GP4, respectively). Each plot contains three rows. For GP1 and GP2 with a row-to-row spacing of 0.40 m, plot dimensions were 1.20 x 0.75 m with (area 0.90 m^2^). For GP3 and GP4 with a row-to-row spacing of 0.25 m, plot dimensions were 0.75 x 1.20 m. Irrigation was applied to maintain sufficient soil moisture in the drought and control fields until flowering had started on 50% of the plots. After that, a period of drought was applied for 3-4 weeks in 2018, and for 6-7 weeks in 2019 to the plots of the drought field. This was achieved by placing mobile rain-out shelters. The irrigation was continued in the control field. After the drought treatment, irrigation was resumed in the drought field and was continued until the late development in control and drought fields.

We evaluated canopy wilting (CW) and leaf senescence (LSEN) symptoms during the period of drought treatment in the drought fields, as described in [23]. CW was scored (score 1-9) three times in 2018 and four times in 2019. LSEN was scored (score 1-9) during and at the end of the drought period. UAV based methodology [126] was used to determine canopy height in each plot using RGB cameras mounted on drones. A growth curve was fitted to the data, and the values of maximum absolute growth rate (AGRmax) and maximum canopy height (CH) were derived for each accession in control and drought fields.

### Phenotypic data analysis

To proceed with the data analysis, filtering was first applied. Plots with less than 30% seedling emergence and outliers identified using Tukey’s rule [127] were removed from the datasets to avoid any bias in analysis. This filtering criteria for seedling emergence was considered sufficient, knowing that on average the emergence was 54-68% in control-drought treatments considering both years 2018 and 2019 (Additional file 1). Data of 2018 and 2019 were considered separately (see [23] for more details). The filtered and cleaned data were analyzed using mixed linear models with the *lmer()* function integrated in the *lme4* package in R [128] using the following base model:

1 $$Y=Intercept +Genotype+Block+Column+Row + Residual$$

Where, *‘Y’* is response variable, *‘Intercept’* represents the overall mean value of the response variable. *‘Genotype’* is a random effect representing the accession, and *‘Block’*, *‘Column’* and *‘Row’* are random effects representing spatial components in the experimental design. *‘Residual’* represents the noise term. The random effects for Block, Column and Row were assumed to be independent, and to originate from an identical, normal distribution. The residuals were also assumed to be independent and identically distributed. *lmer()* uses restricted maximum likelihood (REML) to estimate the variance parameters of the random components (i.e. Genotype, Block, Column and Row).

For each response variable six versions of the base model were tested (considering different combinations of effects) and the output was evaluated using the Akaike Information Criterion (AIC) [129]. The best model was then chosen based on the lowest AIC value. The following choices were made, as reported in [23]:

CW 2018, CW 2019, LSEN 2019, AGRmax 2018, AGRmax 2019, CH 2018, CH 2019:

Y = Intercept + Genotype + Column + Row + Residual

## LSEN 2018:

Y= Intercept + Genotype + Row + Residual

The best linear unbiased predictor value (BLUP) was then calculated for each accession as the sum of the *‘Intercept’* value and the value of the random effect of *‘Genotype’*.

A drought index (Yr) was calculated for the CH and AGRmax data according to [130]:

2 $$Yr= \frac{{\widehat{\mu }}_{c} - {\widehat{\mu }}_{D}}{{\widehat{\mu }}_{c}}$$

Where, ${\widehat{\mu }}_{c}$ and ${\widehat{\mu }}_{D}$ are the estimated BLUPs values of the traits from control and drought treatments respectively. Yr quantifies the strength of the response of each accession to drought stress.

### SNP data

The SNP data included in the present study has been described in [64], where a full description of the methods for genotyping and SNP calling is provided. In short, a total of 477 EUCLEG soybean accessions (including the 359 accessions considered in the present study) were genotyped using the NJAU 350KSoySNP microarray [131]. The same microarray was also used to genotype 394 accessions from the NJAU collection from China. To minimize the chance of misclassification of SNPs, which reduces the false positives in the subsequent analysis, SNP calling was performed on the genotyping data of the EUCLEG and NJAU collections together. The joint analysis provided a total 224,993 SNP markers of high quality spread over the 20 soybean chromosomes with an average distance of 2.6 Kbp between two adjacent SNPs. For the present study we extracted the SNP loci that were polymorphic in the set of 359 EUCLEG accessions. This rendered a set of 139,427 high quality SNPs which are polymorphic at MAF ≥ 0.05.

### Association mapping

The GWAS analysis was performed separately for the SDS and LDS experiments with a Bayesian-information and Linkage-disequilibrium Iteratively Nested Keyway model (BLINK) [132] implemented in the GAPIT R package [133]. BLINK was used with the default settings and LD = 0.2. A threshold value of -Log10 P-value ≥ 4 was set to declare a marker-trait association as significant. The variance explained by each SNP (R^2^) was calculated by applying a simple linear regression between SNP genotype and the phenotype data using *lm()* function in R 3.6.3 [134]. The allele substitution effect of each SNP was calculated by taking the difference in the median value between genotypes carrying the major allele and the minor allele.

### Identification of candidate genes

Candidate regions were delineated around the significantly associated SNPs based on the Linkage disequilibrium (LD) decay distance per chromosome as reported in [64]. LD decay distance per chromosome was calculated in the full EUCLEG collection (477 accessions including the 359 accessions of the present study) and using 224,993 SNPs (including the subset of 139,427 SNPs used in the present study). Candidate regions refer then to the genomic regions surrounding the significantly associated SNPs and spanning up to the LD decay distance calculated for the chromosome where the corresponding SNP is located. A list of genes (genes of interest) located in these candidate regions was retrieved from the soybean reference genome Wm82.a2.v1 using [135]. The corresponding orthologous *Arabidopsis thaliana* genes and their functional annotation were obtained from [65]. [63, 66] and [62] were used to retrieve previously reported QTLs in the candidate regions.

## Supplementary Information

**Additional file 1.**

**Additional file 2.**

**Additional file 3.**

**Additional file 4.**

## Data Availability

All data generated or analyzed during this study are included in this published article [and its supplementary information files].

## Abbreviations

ABA: Abscisic acid
AGRmax-Yr: Drought index for maximum absolute growth rate
BLINK: Bayesian-information and Linkage-disequilibrium Iteratively Nested Keyway model
BLUPs: Best Linear Unbiased Predictors
CH-Yr: Drought index for maximum canopy height
CW: Canopy wilting
GWAS: Genome-wide association study
GST: Glutathione-S-Transferase
LDS: Long duration drought stress experiment
LEA: Late Embryogenesis Abundant
LSEN: Leaf senescence
NDVI: Normalized Difference Vegetation Index
SDS: Short duration drought stress experiment
SNPs: Single Nucleotide Polymorphisms
QTLs: Quantitative Trait Loci
Rab: Response to abscisic acid
ROS: Reactive Oxygen Species
WSC: Water-soluble carbohydrates
WUE: Water use efficiency
