# Supplementary material for: Genetic control of tolerance to drought stress in soybean
Source: BMC Plant Biol. 2022 Dec 28;22:615. doi: 10.1186/s12870-022-03996-w (PMC9795773; doi:10.1186/s12870-022-03996-w)
Supplement: Supplementary file 2 — Additional file 2. [file 12870_2022_3996_MOESM2_ESM.docx]

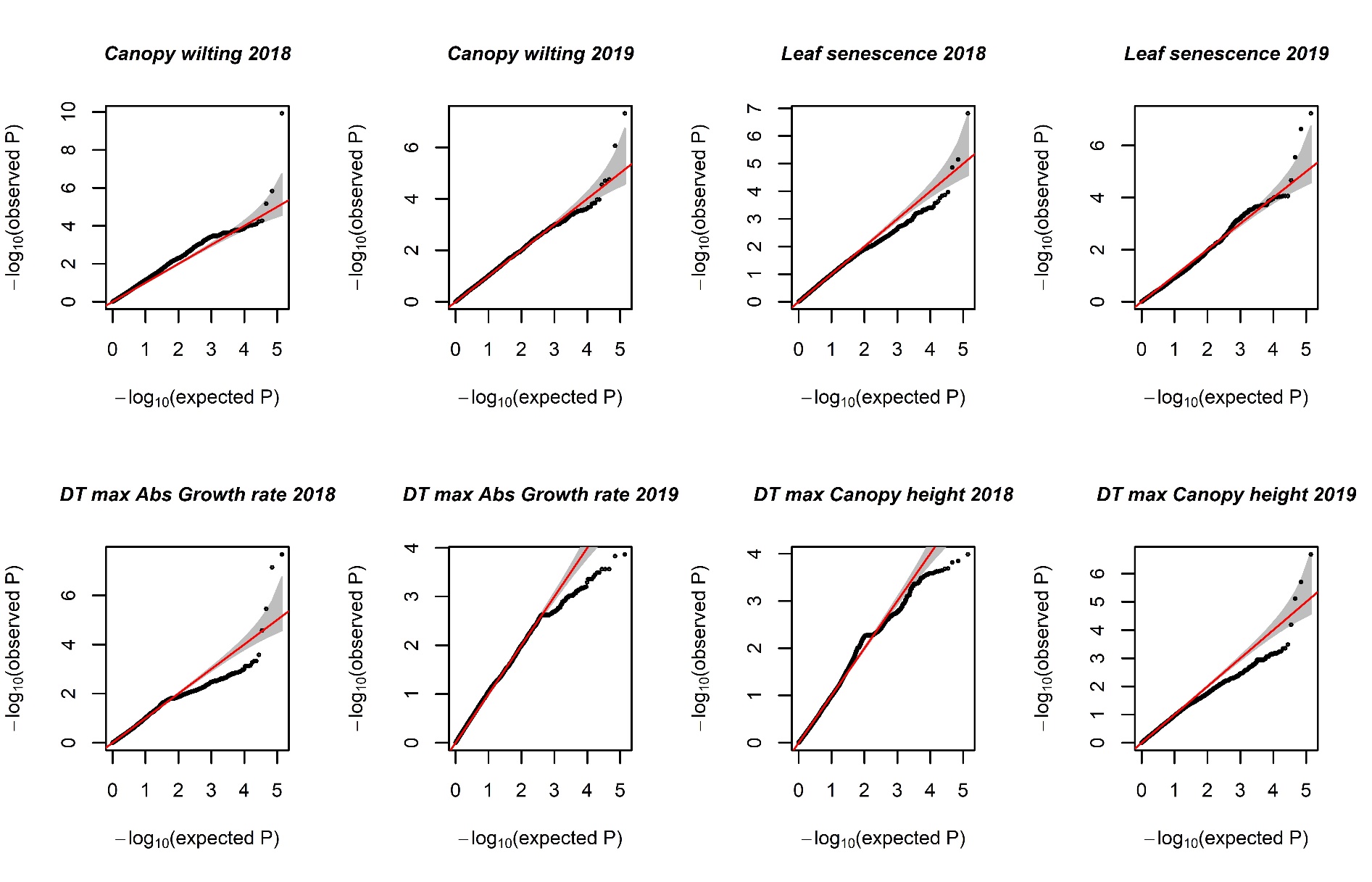


**Figure A1 :** Q-Q plots of GWAS analysis using the BLINK model.


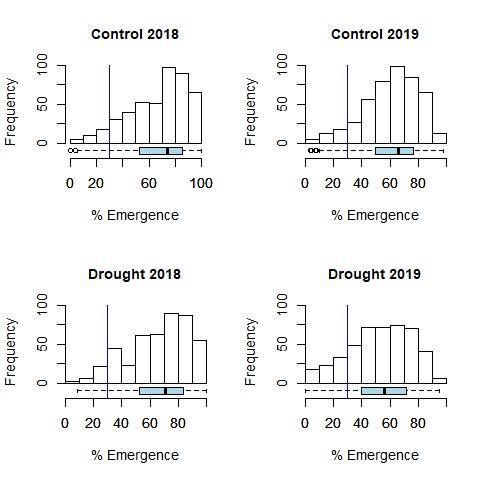


**Figure A2:** Seedling emergence percentage in control and drought treatments of 2018 and 2019. and ‘% Emergence’ on X-axis is the data of seedling emergence percentage and ‘Frequency’ on Y-axis represents the number of plots.
